# Characterization of *qnrB*-carrying plasmids from ESBL- and non-ESBL-producing *Escherichia coli*

**DOI:** 10.1186/s12864-022-08564-y

**Published:** 2022-05-12

**Authors:** Katharina Juraschek, Janina Malekzadah, Burkhard Malorny, Annemarie Käsbohrer, Stefan Schwarz, Diana Meemken, Jens Andre Hammerl

**Affiliations:** 1 Department Biological Safety, German Federal Institute for Risk Assessment (BfR), Max-Dohrn Str. 8-10, 10589 Berlin, Germany; 2 Unit for Veterinary Public Health and Epidemiology, University of Veterinary Medicine, Veterinaerplatz 1, 1210 Vienna, Austria; 3 Institute of Microbiology and Epizootics, Department of Veterinary Medicine, Veterinary Centre for Resistance Research (TZR), Freie Universität Berlin, 14163 Berlin, Germany; 4 Veterinary Centre for Resistance Research (TZR), Freie Universität Berlin, Robert-von-Ostertag-Str. 8, 14163 Berlin, Germany; 5 Institute of Food Safety and Food Hygiene, Department of Veterinary Medicine, Veterinary Centre for Resistance Research (TZR), Freie Universität Berlin, 14163 Berlin, Germany

**Keywords:** *E. coli*, *qnrB*, Fluoroquinolone, Plasmids, *Inc*-group

## Abstract

**Background:**

*Escherichia coli* carrying clinically important antimicrobial resistances [i.e., against extended-spectrum-beta-lactamases (ESBL)] are of high concern for human health and are increasingly detected worldwide. Worryingly, they are often identified as multidrug-resistant (MDR) isolates, frequently including resistances against quinolones/fluoroquinolones.

**Results:**

Here, the occurrence and genetic basis of the fluoroquinolone resistance enhancing determinant *qnrB* in ESBL-/non-ESBL-producing *E. coli* was investigated. Overall, 33 *qnrB*-carrying isolates out of the annual German antimicrobial resistance (AMR) monitoring on commensal *E. coli* (incl. ESBL-/AmpC-producing *E. coli*) recovered from food and livestock between 2013 and 2018 were analysed in detail. Whole-genome sequencing, bioinformatics analyses and transferability evaluation was conducted to characterise the prevailing *qnrB*-associated plasmids. Furthermore, predominant *qnrB*-carrying plasmid-types were subjected to *in* *silico* genome reconstruction analysis. In general, the *qnrB-*carrying *E. coli* were found to be highly heterogenic in their multilocus sequence types (STs) and their phenotypic resistance profiles. Most of them appeared to be MDR and exhibited resistances against up to ten antimicrobials of different classes. With respect to *qnrB*-carrying plasmids, we found *qnrB*19 located on small Col440I plasmids to be most widespread among ESBL-producing *E. coli* from German livestock and food. This Col440I plasmid-type was found to be highly conserved by exhibiting *qnrB*19, a *pspF* operon and different genes of unassigned function. Furthermore, we detected plasmids of the incompatibility groups IncN and IncH as carriers of *qnrB*. All *qnrB*-carrying plasmids also exhibited virulence factors and various insertion sequences (IS). The majority of the *qnrB*-carrying plasmids were determined to be self-transmissible, indicating their possible contribution to the spread of resistances against (fluoro)quinolones and other antimicrobials.

**Conclusion:**

In this study, a diversity of different plasmid types carrying *qnrB* alone or in combination with other resistance determinants (i.e., beta-lactamase genes) were found. The spread of these plasmids, especially those carrying antimicrobial resistance genes against highest priority critically important antimicrobial agents, is highly unfavourable and can pose a threat for public health. Therefore, the dissemination pathways and evolution of these plasmids need to be further monitored.

**Supplementary Information:**

The online version contains supplementary material available at 10.1186/s12864-022-08564-y.

## Background

The spread of antimicrobial-resistant bacteria is a global concern. Commensal *E*. *coli* can acquire and spread various antimicrobial resistance genes sometimes leading to an emergence of multidrug-resistant (MDR) isolates in the livestock, food, and human sector, worldwide. Among antimicrobial-resistant bacteria, extended-spectrum beta-lactamases (ESBL)-producing *E. coli* (ESBL-EC) are of particular concern. While ESBL-EC had become common in hospitalised and healthy people [1], they are also increasingly detected in livestock, food and the community [2]. Their occurrence in livestock and food poses a threat to public health due to a possible transmission to humans via direct contact to colonised animals or the consumption of contaminated food products [1]. Frequently, ESBL-EC carry additional AMR genes, which result in non-wildtype phenotypes against substances of other antimicrobial classes, which can affect an efficient treatment of infections during hospitalization. Prevailing reports highlighted that ESBL-EC isolated from food samples are often associated with increased minimum inhibitory concentrations (MICs) and resistances against avilamycin, colistin, quinolones and fluoroquinolones [3, 4]. Especially, the co-occurrence of genes conferring resistances against quinolones or fluoroquinolones [further designated as (fluoro)quinolones] are of great concern. Fluoroquinolones are classified as highest priority critically important antimicrobials for the treatment of human infections [5], but are also used in the veterinary sector. (Fluoro)quinolone resistance is mainly caused by alterations in the quinolone resistance-determining regions (QRDR) within the *E. coli* chromosome. However, plasmid-mediated quinolone resistance (PMQR) genes are also responsible for a decreased susceptibility or resistance of isolates [6]. As PMQR genes are usually present on mobile genetic elements (MGE) their spread to different bacteria and environments is feasible. Co-localization of PMQR genes and other resistance determinants on MGEs can contribute to MDR development and persistence by co-selection [7]. Interestingly, characterised ESBL-EC were increasingly reported as carriers of *qnr* genes (especially *qnrB*) [8–15] in Europe, the United States, Asia and Africa [16], with different genes and variants of both determinants (*bla* and *qnr*, predominantly *qnrB1* and *bla*_CTX-M-9_, *bla*_CTX-M-3_ or *bla*_SHV-12_) co-localised on the same plasmids. As many different plasmid-types were shown to be responsible for the spread of *qnr* genes, a deeper understanding of their occurrence, diversity and transmission pathways is necessary.

In this study, we investigated the composition of plasmids carrying *qnrB* variants from ESBL-EC and non-ESBL-EC originating from the annual resistance monitoring from livestock and food in Germany. *In-depth* characterization of the plasmids was conducted by phenotypic analyses in combination with whole-genome sequencing (WGS) and bioinformatics analysis of the isolates. The isolates were studied to gain insight into the properties of *qnrB-*carrying MGEs as well as into their transmission potential. Thus, the risk for the spread of MDR *E. coli* outgoing by these *qnrB*-carrying ESBL-EC is discussed.

## Results

### Characteristics of ESBL-/non-ESBL-EC carrying qnrB

Based on available *E. coli* sequences from annual resistance monitoring programs conducted by the National Reference Laboratory for Antimicrobial Resistances (NRL-AR) hosted at the German Federal Institute for Risk Assessment, 33 ESBL (*n* = 29)/non-ESBL-EC (*n* = 4) carrying *qnrB* were identified and subjected to further characterization. The isolates originate from samples of livestock and food taken between 2013 and 2020 and were provided from different German federal state laboratories. In Table 1, essential information on the main characteristics of these isolates is given.

Overall, the 33 analysed *E. coli* were assigned to 26 different 7-locus ST (Supplemental Table 1). We also detected a broad spectrum of different serotypes (*n* = 20), with O89 (*n* = 3), O9 (*n* = 3), O166 (*n* = 2) and O25 (*n* = 2) as the most prominent O-types. However, no MLST-O:H combination occurred twice, underlining the high heterogeneity of the investigated isolates.

Besides the general typing features, the *E. coli* genomes exhibited between 30 up to 79 genes potentially involved in pathogenicity or virulence. The most frequently detected determinants were *chu* gene variants (*n* = 12), *iroN* (*n* = 13), *kpsM* (*n* = 6) and *irp* variants (*n* = 6), *iuc* variants (*n* = 12), *pap* variants (*n* = 4), *pic* (*n* = 1) and *vat* (*n* = 1) genes and *csg*, *fim* variants (including *fimA, B, C, D, E, F, G, H,* and *I*), while *ompA* was present in all isolates.

Antimicrobial susceptibility testing (AST) confirmed non-wildtype phenotypes for the majority of the investigated isolates against the beta-lactam antimicrobials ampicillin, cefotaxime and ceftazidime as well as against the (fluoro)quinolones nalidixic acid and ciprofloxacin for the ESBL-EC. Thus, these isolates exhibited at least resistances against critically important antimicrobials of two different classes. However, a few isolates also exhibited non-wildtype phenotypes against other important antimicrobials like colistin (*n* = 8). The phenotypic data was in good agreement with the AMR genes detected by bioinformatics analysis. In silico*-*typing revealed the presence of multiple AMR genes per isolate (Supplemental Table 1 and Table 1). Most frequently, the beta-lactamase gene *bla*_TEM-1_ and *bla*_TEM-135_ were detected, while *qnrB*19 represented the dominant (fluoro)quinolone resistance determinant*.* Analysing the point mutations within the chromosomal sequence of *gyrA*, *gyrB*, *parC*, *parE*, *pmrA*, *pmrB*, *folP*, 23S/16S rRNA as well as the *ampC* and *rpoB* regions, of which some of the genes are known to be associated with (fluoro)quinolone resistance, we found yet uncharacterised alterations in the above-mentioned sequences within every isolate. For ten isolates, previously characterised point mutations were detected, known to be involved in a decrease of the susceptibility against (fluoro)quinolones. The most frequently appearing point mutations within the *E. coli* are alterations of *gyrA* leading to a decreased susceptibility against nalidixic acid and ciprofloxacin (Table 1). Besides, we detected mutations leading to changes of the amino acid sequence of ParC and ParE also affecting the (fluoro)quinolone susceptibility of the isolates.

To assess a potential transferability of the AMR genes and the association of *qnrB* genes and mobile elements, WGS data of the isolates were used. Potential plasmid sequences of all isolates could be assigned to 24 distinct incompatibility groups (Supplemental Table 2).

**Table 1:** Main characteristics of ESBL-EC (A, *n* = 29) and non-ESBL-EC (B, *n* = 4) associated with *qnrB* from the WGS collection of the NRL-AR. Besides basic metadata (year of sampling and source), information on phenotypic resistance profiles, *in silico*-based prediction of acquired resistance determinants and chromosomal sequence alterations associated with (fluoro) quinolone resistance development as well as the MLST type is given

Isolate	Year of sampling	Matrix (source)	Resistance profile^a^	Acquired resistance determinants^b^	Chromosomal alterations associated with (fluoro)quinolone resistance	multilocus seuqence type (MLST)
13-AB00983	2013	Poultry, meat	AMP, CIP, COL, FOT, STR, TAZ	*acrF, ant(*3*'')-Ia, bla*_EC_*, bla*_SHV-12_*, emrD, lnu(F), mcr*-1.1*, mdtM, qnrB*19	n.d	753
14-AB00641	2014	Poultry, meat	AMP, CIP, COL, FOT, TAZ	*acrF, ant(*3*'')-Ia, bla*_EC_*, bla*_SHV-12_*, emrD, lnu(F), mcr*-1.1*, mdtM, qnrB*19	n.d	48
16-AB00831	2016	Poultry, meat	AMP, CIP, COL, NAL, TET	*acrF, bla*_EC_*, bla*_TEM-1_*, emrD, mcr*-1.1*, mdtM, qnrB*19*, tet(A)*	GyrA (S83L), ParC (S80I), ParE (S458A)	1196
16-AB01284	2016	Poultry, feces	AMP, CHL, CIP, COL, GEN, SMX, TET	*aac(*3*)-IId, aadA*1*, aadA*2*, acrF, bla*_EC_*, bla*_TEM-1_*, cmlA*1*, emrD, mcr*-1.1*, mdtM, qnrB*19*, sul*3*, tet(A)*	n.d	58
16-AB01309	2016	Poultry, feces	AMP, CHL, CIP, COL, FOT, NAL, SMX, TAZ, TET, TMP	*aac(*6*')-Ib-cr*5*, aadA*1*, acrF, arr-*3*, bla*_CTX-M-65_*, bla*_EC_*, bla*_OXA-1_*, bla*_TEM-135_*, catB*3*, cmlA*1*, dfrA*15*, emrD, floR, mcr*-1.1*, mdtM, qnrB*19*, qnrS*1*, qnrS*2*, sul*3*, tet(A)*	GyrA (S83L), ParC(S80I)	2179
16-AB02042	2016	Poultry, meat	AMP, CHL, CIP, COL, GEN, SMX, TET	*aac(*3*)-IIe, aadA*1*, aadA*2*, acrF, aph(*3*'')-Ib, aph(*3*')-Ia, aph(*6*)-Id, bla*_EC_*, bla*_TEM-135_*, catA*1*, cmlA*1*, emrD, mcr*-1.1*, mdtM, qnrB*19*, sul*3*, tet(A)*	n.d	155
16-AB03538	2016	Poultry, feces	AMP, CHL, CIP, COL, SMX, TET	*aadA*1*, aadA*2*, acrF, aph(*3*')-Ia, bla*_EC_*, bla*_TEM-135_*, cmlA*1*, emrD, mc*r-1.1*, mdtM, qnrB*19*, sul*3*, tet(A)*	n.d	48
17-AB00706	2017	Pig, feces	AMP, CIP, FOT, NAL, SMX, TAZ, TET, TMP	*aadA*5*, acrF, aph(*3*'')-Ib, aph(*6*)-Id, bla*_EC_*, bla*_TEM-1_*, catB*3*, dfrA*1*, emrD, mdtM, qnrB*19*, sul*1*, sul*2*, tet(A)*	GyrA (S83L, D87N)	88
17-AB01697	2017	Cattle, feces	AMP, CIP, FOT, SMX, TAZ, TMP	*acrF, bla*_CTX-M-1_*, bla*_EC_*, dfrA*25*, emrD, mdtM, qnrB*2*, sul*1	n.d	657
17-AB02713	2017	Cattle, feces	AMP, CIP, FOT, SMX, TAZ, TET, TMP	*aac(*6*')-Ib-cr*5*, aadA*1*, acrF, aph(*3*'')-Ib, aph(*6*)-Id, bla*_CTX-M-15_*, bla*_EC_*, bla*_OXA-1_*, bla*_TEM-1_*, catA*1*, catB*3*, dfrA*1*, dfrA*14*, emrD, mdtM, mef(C), mph(G), qnrB*1*, sul*1*, sul*2*, tet(A)*	n.d	398
17-AB02827	2017	Pig, feces	AMP, CHL, CIP, FOT, GEN, NAL, SMX, TAZ, TET, TMP	*aac(*3*)-IIe, aac(*6*')-Ib-cr*5*, aadA*1*, acrF, aph(*3*'')-Ib, aph(*6*)-Id, bla*_CTX-M-15_*, bla*_EC_*, bla*_OXA-1_*, bla*_TEM-1_*, catA*1*, catB*3*, dfrA*14*, emrD, mdtM, qnrB*1*, sul*2*, tet(A)*	ParC (A56T, S80I)	744
18-AB00078	2017	Cattle, feced	AMP, CHL, CIP, FOT, SMX, TAZ, TET, TMP	*aac(*6*')-Ib-cr*5*, aadA*1*, acrF, aph(*3*'')-Ib, aph(*6*)-Id, bla*_CTX-M-15_*, bla*_EC_*, bla*_OXA-1_*, bla*_TEM-1_*, catA*1*, catB*3*, dfrA*14*, emrD, floR, mdtM, qnrB*1*, sul*2*, tet(A)*	n.d	154
20-AB00274	2020	Poultry, meat	AMP, CIP, FOT, TAZ, TET	*acrF, aph(*3*')-Ia, bla*_CTX-M-55_*, bla*_EC_*, emrD, mdtM, qnrB*19*, tet(A)*	n.d	1011
20-AB00375	2020	Poultry, feces	AMP, CIP, FOT, NAL, TAZ, TET	*acrF, bla*_EC_*, bla*_SHV-12_*, bla*_TEM-1_*, emrD, mdtM, qnrB*19*, qnrS*1*, tet(A)*	n.d	1626
20-AB00564	2020	Poultry, feces	AMP, CIP, FOT, TAZ	*acrF, bla*_EC_*, bla*_SHV-12_*, emrD, mdtM, qnrB*19	n.d	155
20-AB00569	2020	Poultry, feces	AMP, CIP, FOT, TAZ	*aadA*2*, acrF, bla*_EC_*, bla*_TEM-52_*, emrD, lnu(F), mdtM, qnrB*19	n.d	192
20-AB00611	2020	Poultry, feces	AMP, CIP, FOT, NAL, TAZ	*acrF, blaCMY-2, bla*_EC_*, bla*_TEM-1_*, emrD, qnrB*19	GyrA (S83L), ParC (E84K)	131
20-AB00922	2020	Poultry, feces	AMP, CIP, FOT, NAL, TAZ	*acrF, bla*_CMY-2_*, bla*_EC_*, emrD, qnrB*19	GyrA (S83L)	131
20-AB01255	2020	Poultry, feces	AMP, CIP, FOT, TAZ	*acrF, bla*_EC_*, bla*_SHV-12_*, emrD, mdtM, qnrB*19	n.d	1406
20-AB01339	2020	Poultry, feces	AMP, CIP, FOT, TAZ	*aadA*22*, acrF, bla*_EC_*, bla*_SHV-12_*, emrD, lnu(F), mdtM, qnrB*19*, qnrS*1	n.d	162
20-AB01569	2020	Poultry,feces	AMP, CHL, CIP, FOT, NAL, SMX, TAZ, TET, TMP	*aadA*1*, acrF, aph(*3*'')-Ib, aph(*6*)-Id, bla*_CTX-M-27_*, bla*_EC_*, dfrA*1*, emrD, floR, mdtM, qnrB*19*, sul*1*, sul*2*, tet(A), tet(B)*	GyrA (D87N, S83L), ParC (S80I)	533
20-AB01574	2020	Poultry, feces	AMP, CHL, CIP, FOT, SMX, TAZ, TET, TMP	*aac(*3*)-IIe, aac(*6*')-Ib-cr*5*, aadA*1*, acrF, aph(*3*'')-Ib, aph(*6*)-Id, bla*_CTX-M-15_*, bla*_EC_*, bla*_OXA-1_*, bla*_TEM-1_*, catA*1*, catB*3*, dfrA*14*, emrD, mdtM, qnrB*1*, sul*2*, tet(A)*	n.d	3058
20-AB01775	2020	Poultry, feces	AMP, CIP, COL, FOT, TAZ	*acrF, bla*_EC_*, bla*_TEM-52_*, emrD, mdtM, qnrB*19	n.d	226
20-MO00017	2018	Pig, feces	AMP, CIP, FOT, GEN, SMX, TAZ, TET	*aac(*3*)-IIe, aadA*1*, acrF, aph(*3*')-Ia, bla*_CTX-M-55_*, bla*_EC_*, emrD, mdtM, qnrB*19*, sul*3*, tet(A)*	n.d	10
20-MO00019	2018	Pig, feces	AMP, CIP, FOT, GEN, SMX, TAZ, TET	*aadA*1*, acrF, bla*_CTX-M-1_*, bla*_EC_*, bla*_TEM_*, emrD, mdtM, qnrB*19*, tet(A)*	n.d	10
20-MO00028	2017	Poultry, feces	AMP, CIP, FOT, SMX, TAZ, TET	*acrF, bla*_CTX-M-1_*, bla*_EC_*, emrD, mdtM, qnrB*19*, sul*2*, tet(A)*	n.d	3995
20-MO00045	2017	Poultry, feces	AMP, CIP, FOT, SMX, TAZ, TET	*aadA*2*, acrF, bla*_CTX-M-1_*, bla*_EC_*, emrD, lnu(F), mdtM, qnrB*19*, sul*2*, tet(A)*	n.d	3995
20-MO00078	2018	Poultry, feces	AMP, CIP, FOT, NAL, SMX, TAZ, TET, TMP	*aadA*1*, aadA*5*, acrF, aph(*3*'')-Ib, aph(*6*)-Id, bla*_CTX-M-1_*, bla*_EC_*, bla*_TEM-1_*, dfrA*1*, dfrA*17*, emrD, lnu(F), mdtM, mph(B), qnrB*19*, sul*1*, sul*2*, tet(A)*	GyrA (S83L), ParE (I355T)	4994
20-MO00080	2018	Poultry, feces	AMP, CIP, FOT, GEN, NAL, SMX, TAZ, TET, TMP	*aac(*3*)-IIe, aadA*1*, acrF, bla*_EC_*, bla*_SHV-2_*, bla*_TEM_*, dfrA*1*, emrD, lnu(F), mdtM, qnrB*19*, sul*1*, tet(A)*	GyrA (S83L, D97N), ParC (S80I)	533
14-AB01030	2016	Poultry, feces	AMP, CHL, CIP, COL, SMX, TMP	*aadA*1*, aadA*2*, acrF, bla*_EC_*, bla*_TEM-135_*, catA*1*, cmlA*1*, dfrA*1*, emrD, mcr*-1.1*, qnrB*19*, sul*1*, sul*3*, tet(M)*	ParE (I529L)	131
17-AB00065	2016	Poultry, feces	AMP, CIP, GEN, TET	*aac(*3*)-*VIa*, aadA*1*, acrF, bla*_EC_*, bla*_TEM-1_*, emrD, mdtM, qnrB*19*, tet(A)*	n.d	349
17-AB00089	2016	Poultry, feces	CIP	*acrF, bla*_EC_*, emrD, mdtM, qnrB*19	n.d	69
17-AB00375	2017	Pig, feces	CIP	*acrF, aph(*3*'')-Ib, aph(*6*)-Id, bla*_EC_*, emrD, mdtM, qnrB*19	n.d	117

^a^*AMP* Ampicillin, *CHL* Chloramphenicol, *CIP* Ciprofloxacin, *COL* Colistin, *FOT* cefotaxime, *GEN* Gentamicin, *NAL* Nalidixic Acid, *SMX* Sulfamethoxazole, *STR* Streptomycin, *TAZ* ceftazidime, *TET* Tetracycline, *TMP* Trimethoprim, *n.d.* not detected, *GyrA* Gyrase subunit A, *ParC* DNA topisomerase IV subunit, *ParE* DNA topoisomerase IV subunit.

^b^ If no gene variant is given, the bioinformatics analysis was unable to specify the gene variant

**Table 2:** Incompatibility group (*inc*) prediction based on *inc*-typing of the *qnrB*-carrying contigs and assignment to the best-matched reference plasmid. The frequency of occurrence of *inc* types is provided as absolute numbers in brackets

*inc* prediction based on *qnrB*-carrying contig	Number of isolates	No. of closed plasmids	*inc* group of best-matching reference plasmid	Accession no. of the best-matching reference plasmids
Col440I	24	22	Col440I (*n* = 22)	NC_013782.1 (*n* = 4), NZ_CP039508.1 (*n* = 3), NZ_CP039609.1 (*n* = 11), NZ_CP045445.1 (*n* = 1), NZ_CP045448.1 (*n* = 3)
			ColRNAI (*n* = 2)	NZ_LT985269.1
ColE10	1	1	n.d. (*n* = 1)	n.d
IncN	1	0	IncN (*n* = 1)	NC_019098.1
n.d	7	0	Col440I (*n* = 1)	NC_013782.1 (*n* = 1)
			InH2, IncH2A (*n* = 4)	NZ_CP048350.1 (*n* = 3), NZ_CP024813.1 (*n* = 1)
			- (*n* = 2)	NZ_CP039985.1

*n.d.* not detected

### Characterization of qnrB-carrying plasmids

Among the 33 *qnrB*-carrying *E. coli* isolates, four harboured *qnrB*1, one *qnrB*2 and 28 *qnrB*19. The *in silico* analysis further revealed an association of *qnrB* to contigs of plasmidic origin. Twenty-four isolates harboured *qnrB* on a Col440I replicon carrying plasmid contig. Furthermore, individual plasmid types represented by replicon sequences of the incompatibility groups IncN and ColE10 were detected (*n* = 2). For the remaining isolates (*n* = 7), the *qnrB-*carrying contig could not be associated with a specific replicon type backed in the PlasmidFinder database. Overall, we were able to close the *qnrB*-carrying plasmid genomes of 12 isolates, solely through assembling with unicycler. Interestingly, the majority of those closed plasmids belonged to the Col440I type, while one was assigned to ColE10 (Table 2). We were able to close the remaining plasmids carrying the *qnrB* gene on a Col440I plasmid through primer walking.

For the determination of the *qnrB-*plasmid diversity, the genetic basis of the most prominent types was analysed, including Col440I-, IncN- and IncH2/IncH2A-plasmids.

#### qnrB19 genes on Col440I plasmids

**Fig. 1:**
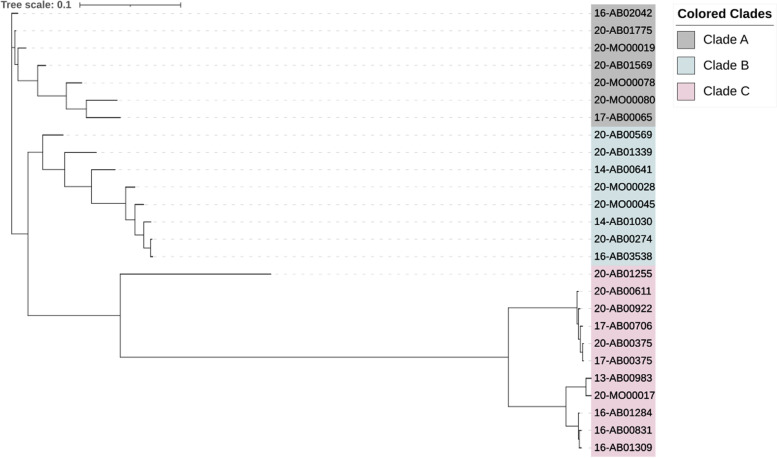
Phylogenetic tree of the Col-plasmids carrying *qnrB*19. The phylogenetic tree was visualised with iTOL v6.3 after creating a nexus file with a multiple Clustal Omega alignment. All tools were used with default parameters

The *qnrB*-associated Col440I plasmids of this study exhibited genome sizes ranging between 2,700 and 3,500 bp with GC-contents of 47.7% to 50.9%. Therewith, we found five reference plasmids representing the different Col440I plasmid-types (Table 2). According to a phylogenetic comparison as shown in Fig. 1, the *qnrB*19 Col440I plasmids clustered into three distinct clades. While clade (A) was only represented by the reference plasmid pEC14-9 (NC_013782), the remaining clades exhibited a slight diversity in their assigned reference genomes. However, Clade (B) was best assigned to the reference plasmid p14-7355.2 (NZ_CP039609), while the most distant clade (C) matched the best to the reference plasmid p3_12888 (NZ_CP045448).

**Fig. 2:**
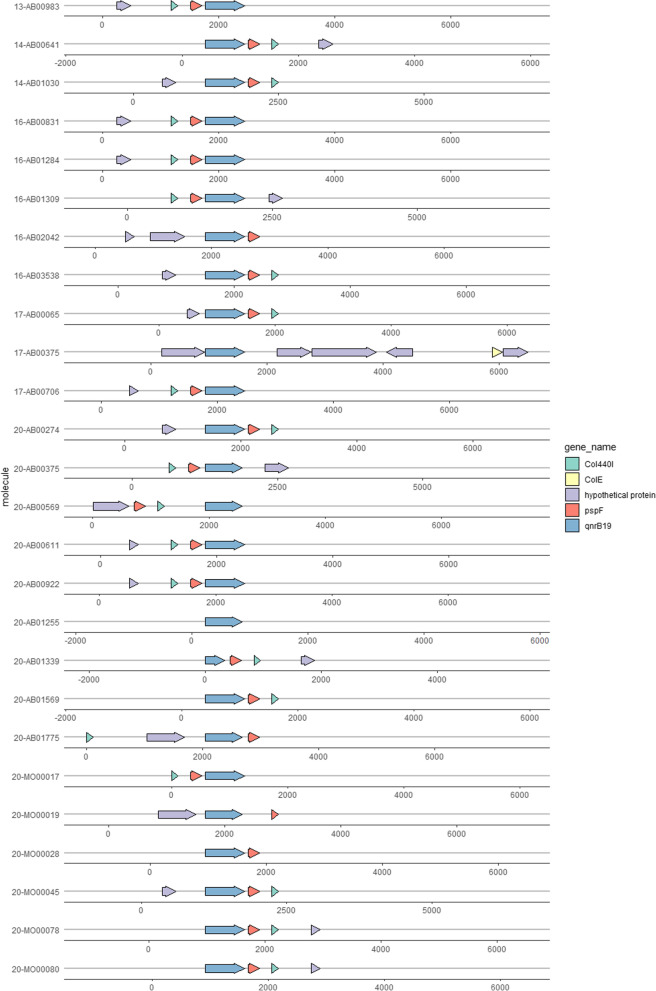
Arrow graph of annotated plasmids assigned to the Col plasmid group, visualised with the ggplot2 package gggenes in RStudio. Genes were aligned according to the position of *qnrB*19*.* The schematic illustration provides no information on the actual plasmid size but the size and position of the annotated genes. The ruler given below the gene map represents an artificial indicator for the size of the region

All Col440I plasmids were equipped with a *pspF* operon transcriptional activator, upstream of the coding sequence (CDS) of the pentapeptide-encoding gene *qnrB*19. Close to the replication initiation protein, the gene for the transcription factor *Sp1* was identified, as well as a putative CDS of a yet uncharacterised function (Fig. 2). The main differences of the phylogenetic clusters are caused by the occurrence of these sequences encoding proteins of unknown function. On the Col440I genomes, no further AMR, virulence, or biocide resistance genes were detected. Interestingly, these plasmids also lack insertion sequences potentially associated with a further dissemination of the *qnrB* gene. In Fig. 2, the genetic diversity of the Col440I-plasmid is shown. Furthermore, the organization and function of the CDS predicted on the Col440I-genomes are given. Based on the prevailing data a core genome of this plasmid type can be assigned to the presence of *qnrB*19 and *pspF* gene.

By analysing the Col440I genomes with the in silico mob-suite tool, the plasmids were assigned to be mobilizable. In general, the origin of transfer (*oriT*) of the plasmids was subjected to the conjugative transfer system of the MOB_P_ type, but no type IV secretion system (T4SS) was detected. Thus, the plasmid is transmissible by a helper plasmid but not self-transmissible. However, in vitro studies using the *E. coli* isolates for filter mating with the sodium acid-resistant *E. coli* strain J53, yield negative results under double selective conditions (NAL/SAC), suggesting that the plasmid could not be transmitted due to a lack of a suitable helper plasmid or based on missing determinants or altered sequences involved in mobilization.

#### Characterization of IncN plasmids carrying qnrB2

Out of the prevailing *qnrB* collection, only one ESBL-producing *E. coli*, from cattle, represented a *qnrB*2 determinant. This one was located on an IncN plasmid. *qnrB*2 was identified on a 39,563 bp contig, which could not be circularised to a complete plasmid genome using unicycler alone. However, reference-based mapping with plasmidID against a complete plasmid database revealed a close relationship (92.33% reference coverage) to the *E. coli* plasmid pHHA45 (NC_019098). This plasmid represents an IncN plasmid of 39,510 bp with a GC content of 50.5%. A sequence comparison of both plasmid sequences revealed the presence of repetitive sequences on pHHA45, which might be responsible for failing circularisation by using the unicycler program.

**Fig. 3:**
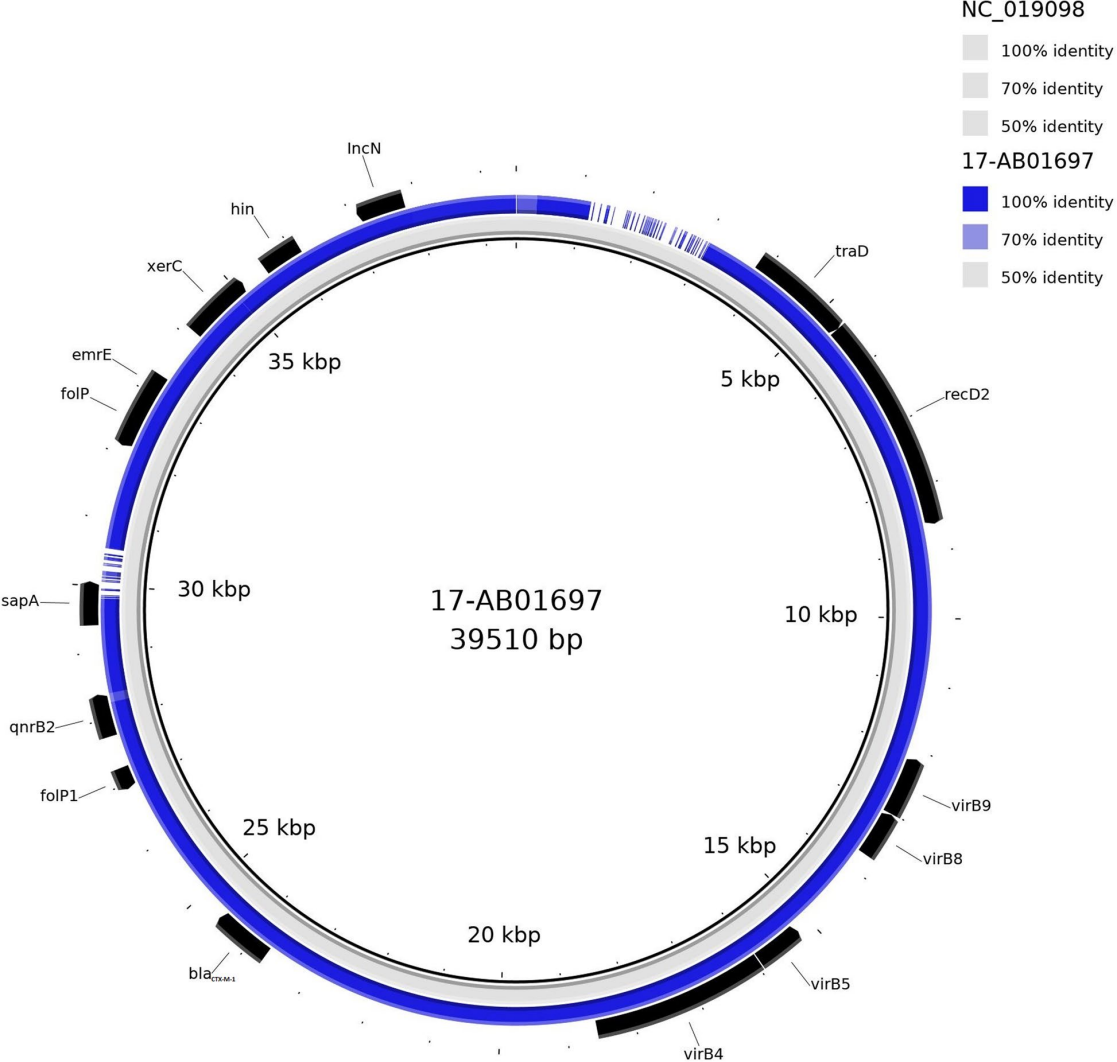
IncN plasmid contigs mapped against the best matching reference NC_019098 with BRIG

However, the derived contigs of the isolate comprised all essential information for the evaluation of the impact of this IncN plasmid type. In proximity to *qnrB*2, the plasmid carried a *bla*_CTX-M-1_ gene, which is also present on the reference plasmid pHHA45 (NC_019098) (Fig. 3). The *bla*_CTX-M-1_ gene is flanked by IS*26* elements. Upstream to *qnrB*2, the dihydropteroate synthase (encoded by *folP*) and *bla*_CTX-M-1_ flanked by IS*26* elements are located. Downstream of *qnrB*2, *sapA* encoding a peptide transport periplasmic protein and a further dihydropteroate synthase was identified. Moreover, the multidrug transporter gene *emrE*, associated to the transposase IS*Ssu9*, was located on this plasmid. The mob-suite analysis predicted that the IncN plasmid is self-transmissible (conjugative). In silico analysis revealed the presence of MOB_F_ relaxase and the MpfT mating pair formation (*mpf*) system. The predicted self-transmissibility of the plasmid could be confirmed by *in vitro* filter mating studies. We observed a transmission rate of 10^5^ to 10^6^ per donor cell.

#### Characterization of IncH2/IncH2A plasmids carrying qnrB1

**Fig. 4:**
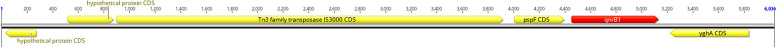
Organization of the *qnrB*1 coding region on the IncH plasmids. The region represents the contig as detected for the four investigated *qnrB1* carrying *E. coli* of our study. This contig, bioinformatically mapped to the reference plasmids containing the IncH replicon sequence, did always consist of the same structure as presented here

Four of the investigated *E. coli* revealed plasmid sequences associated with *qnrB*1 and IncHI2-IncHI2A replicon sequences. Here, we were able to assign contigs, bioinformatically clustering together, from our isolates to a reference plasmids, carrying the *qnrB1* as well as the IncH replicon sequence on the same plasmid. Therewith, these contigs of our isolate fully covered the *qnrB1* and IncH replicon sequence region. Two of the isolates were recovered from cattle, one from pig and one from poultry. De novo assemblies yielded *qnrB*1-carrying contigs of 6 and 12 kb for three and one isolate, respectively. Using the plasmidID tool we were able to identify p23_A-OXA140 (NZ_CP048350; 99% coverage) and pCRENT-193_1 (NZ_CP024813; 99% coverage) as the closest relatives. The respective reference plasmids ranged between 279 and 298 kb in size and exhibited GC contents of 48%. Both reference plasmids are 92% identical at nucleotide level to each other. Here, *qnrB*1 was located downstream to the *pspF* and a Tn*3* family transposase gene (Fig. 4).

Here, the IS*6* family transposons (IS*26*) flanked an area, containing the AMR genes *qnrB*1, *aac*(3)-lle, *aac*(6')-Ib-cr5 and *bla*_OXA-1_. All AMR genes detected on these plasmids are located in a region of 40 kb. in silico analysis revealed a self-transmissibility of the plasmid due to the detection of the Mpf_T_ system and the MOB_H_ relaxase type. The plasmid pCRENT-193_1(NZ_CP024813) carries multiple AMR genes like *aac*(3)-IIe, *aac*(6')-Ib-cr5, *aadA*1, *aph*(3'')-Ib, *aph*(6)-Id, *bla*_CTX-M-15_, *bla*_OXA-1_, *bla*_TEM-1_, *catA*1, *catB*3, *dfrA*14, *qnrB*1, *sul*2 and *tet*(A). The same genes were detected in the WGS data of 18-AB00078 analysed in this study. A similar resistance gene profile, only lacking *aadA*1 and *catA*1, was detected on the p23_A-OXA140 genome (NZ_CP048350). However, *aadA*1 and *catA*1 were present in the respective matching plasmids from our investigated isolates and resembled to the reconstructed *qnrB*1 plasmid. Furthermore, we detected the *hipA* gene, coding for a serine/threonine-protein kinase toxin, which is the toxic component of a type II toxin-antitoxin (TA) system. The binding partner HipB encoding gene was not detected in proximity of *hipA* and could not be identified in the complete WGS dataset representing the complete *E. coli* isolate. We also detected the tellurium ion resistance gene *terC*, often associated with pathogenic bacteria [17], in the investigated plasmids. Overall, all *qnrB*-carrying plasmids were shown to be self-transmissible under natural condition based on the observed transfer rates of 10^2^ to 10^4^ per donor cell.

## Discussion

### Characteristics of ESBL and non-ESBL E. coli carrying qnrB-plasmids

In this study, 33 ESBL-/non-ESBL-EC carrying *qnrB* on an extrachromosomal element were characterised in detail. This co-occurrence of ESBL and (fluoro)quinolone resistance genes in *E. coli* poses a threat to public health, as these antimicrobials are highest priority critically important substances in human medicine.

Among the investigated isolates, a broad variety of sources of *qnrB*-carrying *E. coli* was found. While poultry seems to be the predominant source for *qnr* genes [18], plasmids with PMQR were also found in other sources. The presence of *qnrB* in ESBL-EC from poultry is frequently reported. The latest summary report of the European Food Safety Authority (EFSA) on AMR in zoonotic and indicator bacteria from humans, animals and food [18] also addresses this trend. Although the incidence of ESBL-EC was generally low, it was most often detected in broiler isolates (Member State group level of up to 30%). However, EFSA further reported a high level of ciprofloxacin- and nalidixic acid-resistant *E. coli* especially from broilers (median 73.5% for ciprofloxacin and 64.1% for nalidixic acid) and turkeys. Thus, poultry seems to be a common reservoir for ESBL-EC and (fluoro)quinolone-resistant *E. coli*. General, the occurrence of MDR *E. coli*, as characterised in this study, along the food chain poses a risk for a transmission of these bacteria to human via food products.

Interestingly, *qnrB*-positive ESBL-EC were also detected among isolates of the international high-risk clone ST131 [19] and O89 serotype. ST131 isolates are known to represent a predominant sequence type among extraintestinal pathogenic *E. coli*, which comprise ESBL-positive as well as (fluoro)quinolone-resistant isolates [20]. *E. coli* of the serotype O89 are often associated with MDR [21]. Based on the results of this study, a similar association was observed for these *E. coli* types. The occurrence of *qnrB*-carrying plasmids in various STs of ESBL-EC further demonstrated that these plasmids exhibit a broad adaptability to *E. coli* of different ST.

It had been discussed before, that *qnrB* represents the dominant PMQR group in humans, while *qnrS* seemed to be more frequent in the environment [22], the veterinary and food sector [23–26]. This emphasises the need for a better understanding of the composition and impact of *qnrB*-carrying plasmids to estimate the transmission possibilities from animal to humans.

Here, the ESBL-EC were mostly phenotypically resistant against penicillins, cephalosporins and (fluoro)quinolones. Further, every isolate carried a *bla* gene coding for different TEM- or less frequently CTX-enzyme variants. For *qnrB*, the variant *qnrB*19 seemed to be predominant. Interestingly, some isolates showed genes encoding penicillinases and ceftazidime resistance. This observation needs to be further verified and characterised in detail to determine, if also other mechanisms like mutations in PBP3 and efflux pumps can cause these effects. However, all isolates of this study further exhibited phenotypical resistance to antimicrobials of other classes and were shown to carry various AMR genes co-occurring in the same isolate.

The detected virulence factors (*ompA*, *csg*, *fim, chu*, *iroN*, *kpsM*, *irp*, *iuc*, *pap*, *pic* and *vat*) may contribute to an increase in the pathogenic potential of these *E. coli*. The outer membrane protein A (OmpA) contributes to pathogenesis. The capsular antigen (KpsM) represents a protection factor against phagocytosis. The siderophore aerobactin gene (*iuc*) as well as *irp, iroN* and *chu* are associated with iron uptake often present in uropathogenic *E. coli* (UPEC). The genes *pap* (coding for P fimbriae), *fim* (type 1 fimbriae) and *csg* (curli fibers) contribute to the adhesion properties of the *E. coli.* The serine protease autotransporter encoding gene *pic* and the vacuolating autotransporter encoding gene *vat* do represent toxins. All detected factors individually contribute to an increase in the pathogenic potential of *E. coli* [27]. The presence of these virulence factors, in *qnr*-carrying ESBL-EC demonstrates an aggregated risk. As virulence factors are also frequently present on plasmids [28], their potential spread can increase the clinical impact of the bacterium dramatically. Further subtyping results, i.e., the phylotype and detected *fim* variants are given in Supplemental Table 3.

While PMQR genes are the main contributors for horizontal (fluoro)quinolone resistance transmission, alterations in the sequences of the DNA gyrase and topoisomerase IV genes are the main reason for resistance against (fluoro)quinolones in *E. coli* [29]. We detected previously determined single nucleotide polymorphisms (SNPs) leading to high-level resistances within ten of 33 isolates. The SNPs where mainly identified in the genes *gyrA* and *parC*. Especially the mutations in the S83L (in GyrA) and D87N (in ParC), as found here, are common [30, 31]. These mutations, in combination with the carriage of a *qnr* gene, are responsible for the (fluoro)quinolone resistance phenotype [32]. However, we further detected yet uncharacterised SNPs in the QRDR for every analysed isolate (Supplement Table 4). As the study only focused on (fluoro)quinolone-resistant *E. coli*, it might be possible that these mutations also contribute to the observed (fluoro)quinolone resistance. Another appropriate interpretation might be a higher contribution of the *qnrB* genes to (fluoro)quinolone resistance, than commonly expected [33].

### Prevalent qnrB-carrying plasmids in ESBL-/non-ESBL-EC

We identified a 39.5 kb IncN plasmid carrying *qnrB*2 in combination with *bla*_CTX-M-1_ surrounded by IS*26* elements as previously described [34, 35]. The *bla*_CTX-M-1_ in proximity to IS*26* elements was also detected on other plasmid types, suggesting that transmission of this specific region took place via IS*26*-mediated transfer [36]. The *folP* gene, identified upstream of the *qnrB*2 gene, is another characteristic of this plasmid. The *folP* gene encodes the dihydropteroate synthase (DHPS) enzyme, which is usually encoded on the chromosome and represents the target of sulfonamides [37, 38]. The presence of *folP* on the plasmid may represent a genetic advantage for *E. coli*, as it ensures the folate biosynthesis pathway. Furthermore, the MDR transporter, encoded by *ermD* was detected. It represents a small MDR transporter known to confer resistance to a broad spectrum of disinfectants and quaternary cation compounds [39]. The gene *ermD* was detected close to the IS*Ssu9*, also suggesting IS-mediated transfer. In this study, we isolated the plasmid from bovine *E. coli*. However, similar IncN plasmids were identified in isolates of various sources (food, livestock and humans) from the Czech Republic, Poland, Denmark and Italy [40]. Dolejkska et al. [41] described comparable plasmids also carrying a *bla*_CTX-M-1_, in addition to *qnrS*1 or *qnrB*19, but to the best of our knowledge not together on the same IncN plasmid. As this plasmid was determined to be conjugative as well as to be a broad-host range plasmid [40], the risk resulting from this special IncN plasmid and the evolvement of AMR gene accumulation should be further monitored. Another detected plasmid type in this study were plasmids of the IncHI2-IncHI2A incompatibility group. We detected this plasmid type in association with a *qnrB*1 gene in isolates of different animal sources, which suggests a possible broad dissemination. We identified multiple AMR genes on our *qnrB*1-carrying plasmids. All IncH plasmids from this study exhibited the following AMR genes: *aac*(3)-IIe*, aac*(6')-Ib-cr5*, aadA*1*, aph*(3'')-Ib*, aph*(6)-Id*, bla*_CTX-M-15_*, bla*_OXA-1_*, bla*_TEM-1_*, catA*1*, catB*3*, dfrA*14*, qnrB*1*, sul*2 *and tet*(A), leading to resistance against aminoglycosides, beta-lactams, chloramphenicol, trimethoprim, (fluoro)quinolones, sulphonamides, and tetracyclines. IncH-like plasmids were previously reported as accumulators, carriers and spreaders of various resistances [42]. The carriage of multiple AMR genes, as present for this plasmid type, probably presents a risk when transmitted. The co-occurrence of genes conferring resistance against two broad antimicrobial classes is alarming. With the presence of *bla*_CTX-M-15_, *bla*_OXA-1_ and *bla*_TEM-1_, three different beta-lactamase genes were located on the same *qnrB*1-carrying plasmid. The IS*26* element in proximity to *qnrB*1 and *bla*_OXA-1_ is known to be responsible for spreading multiple AMR genes [43]. Varani et al*.* described the importance of IS*26* in clinical settings. They mentioned an increased frequency of plasmids carrying IS*26* involved in aggregation of antimicrobial resistance genes [43]. Harmer and colleagues described IS*26* as key element for the dissemination of AMR genes in Gram-negative bacteria [44]. We further detected *hipA* on the IncH plasmid. We were not able to detect the antitoxin component *hipB*, neither in other plasmids nor in the chromosomal DNA of the respective isolates. Thus, it remains unclear how the *E. coli* copes with the burden of the toxin produced from *hipA*. We can assume that the presence of this component is a benefit for the plasmid stability within the isolate. The detected *terC* virulence factor on the plasmid is quite common for IncH plasmid types. It was described as responsible for the control of resistance to infections by some bacteriophages [45]. Thus, it may confer another advantage for the host to retain the respective plasmid. Further, IncH-type plasmids were often detected in animal and human isolates and seemed to be disseminated among different sources contributing to the spread of AMR genes from animals to humans or vice versa [45]. As we determined the self-transmissibility of the IncH-like plasmids characterised within this study, a possible spread of this plasmid carrying multiple AMR genes is indeed given.

The most frequently detected *qnrB* plasmid type in the ESBL-/non-ESBL-EC in this study belonged to the Col440I-like group. All Col440I plasmids carried a *qnrB*19 gene and a *pspF* operon, as well as the gene for the transcription factor *sp1*. The protein sequence of the hypothetical protein as well as the non-coding regions altered within the different clusters. With the detection of the *mob*_*P*_ relaxase gene, this plasmid was categorised as mobilizable, but not self-transmissible. The same plasmid (100% identity) has been described before with exactly the same genome structure but was assigned to a different plasmid type. Karczmarczyk and colleague identified this “ColE-like” plasmids from food samples in Colombia to carry *qnrB*19 [46]. Pallecchi et al*.* characterised the same structure as ColE-like plasmid. They found this small *qnrB*19-carrying plasmid in *E. coli* from humans around Latin America with a high frequency and suggested a major role of this small plasmid in *qnrB* dissemination. As the plasmid is small and contains only a few genes, the authors hypothesised that it could have undergone a subsequent excision [47]. This was supported by other studies, describing *qnrB*19 within a comparable genetic environment in larger plasmids, associated with IS*Ecp1C*-based transposons [48]. Moreno-Switt et al*.* [49] described that this small *qnrB*19-carrying plasmid was reported in Europe, the U.S.A. and South America in *Salmonella* obtained from food, animals and humans. They demonstrated how this *qnrB*19-carrying plasmid type was transmitted between different *Salmonella* serotypes through a P22-mediated transduction, probably explaining the frequent detection of this small plasmid. Although we only detected *qnrB*19 on the small Col440I plasmids, there are some studies available, presenting the *qnrB*19 on different plasmids also containing *bla*_TEM-1_ or *bla*_SHV-12_ [50]. Blasting the Col440I plasmid against the NCBI database, we detected an 11.3 kb plasmid (FDAARGOS_1249) containing the backbone of the *qnrB*19-carrying plasmid but also additional genes, like the plasmid mobilization gene encoding MOB_C_.

Overall, we were able to thoroughly determine and characterize the structures of the plasmids carrying the *qnrB* gene. However, as we investigated the isolates with short-read sequencing the limitations for closing these plasmids has to be mentioned. Although, in silico estimation of the whole plasmid from short reads is getting more reliable, an optimised approach would include long-read sequencing of the plasmids of interest. As previously shown, especially for the determination of large plasmid genomes long read sequencing is necessary [51]. Due to the occurrence of mobile genetic elements or repetitive sequences, short read sequencing techniques represent limitations for addressing this issue.

### Risk posed by qnrB-carrying plasmids in ESBL-/non-ESBL-EC

We detected different *qnrB* genes on plasmids within the *E. coli* isolates of this study. All investigated isolates were resistant to (fluoro)quinolones. Usually, plasmidic factors were accounted only with a decrease of the susceptibility of the isolate not necessarily resulting in a non-wildtype phenotype of the isolates. However, not all isolates carried a known mutation within the respective QRDRs. Thus, it might be possible that the presence of a *qnrB* gene without any other yet characterised chromosomal alteration in the PMQR or the presence of other plasmidic factors can lead to a resistance phenotype for (fluoro)quinolones. Different studies had already explained, how the *qnr* genes are able to alter the resistance against (fluoro)quinolones due to mutations in the chromosomal QRDR regions and how the presence does allow other antimicrobial resistance genes to enter and persist. Thus, Li and colleagues reported on how QnrB promotes DNA replication stress that leads to an increased bacterial mutation risk. In their investigations, they measured a two-fold increase in the mutation rate, when QnrB is expressed. Further, they found how QnrB is responsible for the accumulation of mutations, also including quinolone resistance mutations. Overall, they suggested that QnrB could be in charge for promoting the persistence of plasmids leading to resistance against respective antimicrobial agents [52]. These results let us assume, that the presence of *qnrB* genes in our isolates are also responsible for an environment allowing some mutations to occur and therewith promote the presence of different resistance profiles.

When the presence of *qnrB* was investigated for the first time in-depth, the observation of its association with ESBL-producing bacteria was mentioned [53]. Jacoby et al. explained how the *qnrB-*carrying isolates, primarily detected in the U.S.A. and India, were always present with *bla*_SHV-12_ or *bla*_CTX-M-15_ genes on the same plasmid [53]. The combined presence of extended-spectrum beta-lactamases encoding *bla* and *qnr* genes together within the same isolate, and/or on the same plasmid, is highly unfavourable for medical treatment. Both genes are able to confer resistance against two important classes of antimicrobial agents. Their spread to different sources, as different bacterial species, different environments or to humans is a high risk. Kawamura et al. described how ESBL-EC have become common among healthy people worldwide. They explained how most ESBL-EC usually acquired co-resistance to (fluoro)quinolones and other clinically important antimicrobial agents [1]. As we detected the ESBL and non-ESBL-EC, carrying resistance determinants against (fluoro)quinolone within isolates recovered from livestock and food, one could assume, that the respective plasmids had spread over different areas, thus, demonstrating the necessity of a One-Health approach when estimating the risk especially arising from *qnr*-carrying ESBL-EC and from non-ESBL-EC.

## Conclusion

In this study, we described and analysed the presence of different ESBL-/non-ESBL-EC carrying a *qnrB* resistance gene. We found *qnrB*1 *and qnrB*2 genes to be present on larger plasmids, carrying multiple antimicrobial resistance genes, including different *bla* genes (e.g. *bla*_*CTX-M-15*_, *bla*_*TEM1*_, *bla*_*OXA-1*_). Further, we found *qnrB*19 in particular on small Col440I plasmids. The presence of these PMQR genes together with extended-spectrum beta-lactamases encoding different *bla* genes in the same isolate or even on the same plasmid harbors a risk for public health. Especially the small Col440I plasmids seem to play an important role in the dissemination of *qnrB*19 genes, as they frequently had been described in *E. coli* and other *Enterobacteriaceae* from various sources and areas. The spread of MDR conferring plasmids contributes to an impaired treatment possibility. Thus, their evolution should be studied further, especially with an One-Health approach.

## Methods

### Isolate selection

For this study, *E. coli* collected during the German AMR monitoring of commensal (ZoMo-monitoring) and ESBL-/AmpC-producing (ESBL-monitoring) from food and livestock were chosen, as directed by the Federal Office of Consumer Protection and Food Safety (BVL). In addition, isolates carrying *qnrB* genes from research projects (indicated in the designation of MO) were added. Characterization of the isolates was based on WGS data obtained from Illumina NextSeq sequencing. ESBL-/non-ESBL-EC carrying *qnrB* genes were further investigated here. In total 33 epidemiologically unrelated *E. coli*, carrying *qnrB* were included. All isolates were initially cultivated on lysogeny broth [Luria Broth Base (Miller's LB Broth Base), Invitrogen-Thermo Fisher, Darmstadt, Germany] for 16–18 h at 37 °C.

### Antimicrobial susceptibility testing

Antimicrobial susceptibility testing (AST), of *E. coli* was conducted by determining minimum inhibitory concentrations (MICs) (including sulfamethoxazole, trimethoprim, ciprofloxacin, tetracycline, meropenem, azithromycin, nalidixic acid, cefotaxime, chloramphenicol, tigecycline, colistin, ampicillin, gentamicin and ceftazidime). Broth microdilution was performed according to EUCAST recommendations on a standardised European antimicrobial test panel (EUVSEC/EUVSEC2; Sensititre™, TREK Diagnostic Systems, UK). Therewith, all antimicrobials were tested in ranges as given by the European Commission Implementing Decision No. 2013/652/EU [54]. The results were interpreted according to EUCAST epidemiological cut-off values (ECOFFs) [55]. For quality assessment during MIC evaluation, the *E. coli* isolate ATCC 25,922 was included in every measurement. An ESBL-phenotype was assigned when the following phenotypic patterns were detected: cefotaxime or ceftazidime > 1 mg/L and meropenem ≤ 0.12 mg/L and cefoxitin ≤ 8 mg/L and no combination of cefotaxime/clavulanic acid and/or ceftazidime/clavulanic acid. Strains were further determined as phenotypically resistant against (fluoro)quinolones when expressing a MIC of 32 mg/L for nalidixic acid and/or 0.12 mg/L for ciprofloxacin. For better monitoring, we chose all isolates representing these criteria or one MIC step lower.

### Whole-genome sequencing

For conducting whole-genome-sequencing (WGS), genomic DNA of *E. coli* was extracted using the PureLink Genomic DNA Mini Kit (Invitrogen-Thermo Fisher) according to the manufacturer’s recommendation. The library for sequencing purposes was generated with the Nextera DNA Flex Library Preparation Kit (Illumina®, San Diego, CA, USA) as previously described by Borowiak et al. [56]. Short-read sequencing was performed in paired-end WGS mode in 2 × 151 cycles with the Illumina® NextSeq™ 500/550 Mid Output Kit v2.5 (300 Cycles). If appropriate for plasmid closing, a primer-walking approach was applied. Therefore, the gDNA was used as template with primers (Supplement Table 5) derived from the assembled plasmid genomes. The amplification was performed in a Bio-Rad Thermal Cycler (Bio-Rad, Feldkirchen, Germany). The purification of the PCR amplification products was performed using the illustra GFX PCR DNA and Gel Band Purification Kit (Cytiva Europe, Freiburg, Germany). Sanger sequencing was performed by Eurofins Genomics (Eurofins Genomics, Ebersberg, Germany).

### Bioinformatics analysis

Raw reads were trimmed with Aquamis [57] and de novo assembled using unicycler [58]. Quality assessment of the assemblies was conducted with quast [59]. Analysis of virulence factors, antimicrobial resistance genes, serotype and plasmid markers was conducted with bakcharak [60]. The prediction of the 7-locus MLST for each *E. coli* was screened with the tool v0.2 (https://github.com/tseemann/mlst) [61]. The annotations of the generated fasta files were achieved with prokka v1.14.5 [62]. To estimate the regions of local similarity between sequences BLAST was utilised (https://blast.ncbi.nlm.nih.gov/Blast.cgi). We used the PointFinder tool v2.2 [63] for detecting alterations in the chromosome responsible for (fluoro)quinolone resistance in *E. coli*. The refSNPer tool v1.0.0 was used as described before [64] to find best matching reference plasmids. To confirm the estimation, we screened our contigs, whether they were assignable to the estimated reference plasmid. We conducted this screening with the gplas tool v0.6.0. The gplas tool bins the respective contigs and therefore allows accurate plasmid-prediction [65]. To visualize and confirm these findings we used the mapping-based and assembly-assisted plasmid identification tool plasmidID v1.6.5 (https://github.com/BU-ISCIII/plasmidID) with a plasmid database containing all available closed plasmids from NCBI. For detecting all present MGEs within one isolate, the MGEfinder v1.0.6 was used [66]. For estimating the conjugative properties of the isolates, through detection of the MOB and mpf determinants, mob-suite v3.0.1 was utilised [67]. To detect the presence of Toxin-Anti-Toxin systems, we used the sling tool v2.0 [68]. For estimation of the phylotype, the phylotyper superphy (https://github.com/superphy/insilico-subtyping) was used. Finally, BRIG was used for visualizing the circular comparison between genomes [69]. For generating and visualizing the phylogram, Clustal Omega was used to generate the newick file [70]. This format was then used in iTOL [71]. Further, the alignment was visualised in geneious [72]. An arrow-based alignment was created with the gggene v0.4.1 extension of ggplot2 in R (https://wilkox.org/gggenes/). If not mentioned otherwise, all tools were used with default parameters.

### *in vitro* filter mating studies

The transferability of the *qnrB*-carrying plasmids was determined by filter mating studies on solid LB agar. Liquid cultures of donor and recipient bacteria were mixed in a ratio of 1:2 (500:1000 µl), centrifuged at 5,000 × g for 5 min. Thereafter, the supernatant was discharged, and the pellet was resuspended in 150 µl LB broth, applied on a 0.2 µm pore-size filter on an LB agar plate and subjected to incubation at 37 °C. After 24 h, bacteria were resuspended from pore-size filters in 4 ml LB broth and 100 µl of the bacterial suspension was applied on double selective agar plates [nalidixic acid (NAL) 8 mg/L and sodium acid (SAC) 100 mg/L]. After 24 h of incubation at 37 °C, the plates were interpreted for presumptive transconjugants. To confirm successful plasmid transmission, the transconjugants were subcultivated on double selective LB agar (NAL/SAC) and further analysed for the presence of the *qnrB* gene by PCR.

## Supplementary Information

**Additional file 1: Supplement Table 1.** In silico detected ST, O:H, resistance genes and gene variants of every analysed isolate. **Supplement Table 2.** In silico detected plasmids marker for every analysed isolate. **Supplement Table 3.** Phylotype and fim variants of investigated isolates. **Supplement Table 4.** PointMutation within isolate, determined with PointFinder. **Supplement Table 5.** Nucleotide Sequence and Amplification Temperature of special designed Primers for Primer-Walking. **Supplement Table 6. **Accession Number of submitted plasmid fasta files.

## Data Availability

All data generated or analysed during this study are included in this published article and its supplementary information files. The datasets used and analysed during the current study are available from the corresponding author on reasonable request. Contigs representing the detected *qnr*-carrying plasmid are available under the project number PRJNA755260 in NCBI. Accession Numbers are presented in Supplemental Table 6.

## Abbreviations

(fluoro)quinolones: Fluoroquinolones and quinolones
AMP: Ampicillin
AMR: Antimicrobial resistance
BLAST: Basic local alignment search tool
cg: Core genome
CHL: Chloramphenicol
CIP: Ciprofloxacin
COL: Colistin
DNA: Deoxyribonucleic acid
*E.*: *Escherichia*
ECOFF: Epidemiological cut-off values
EFSA: European Food Safety Authority
ESBL: Extended-spectrum β-lactamase
ESBL-EC: Extended-spectrum β-lactamase-producing *E. coli*
EU: European Union
FOT: Cefotaxime
GEN: Gentamicin
MGE: Mobile genetic element
MIC: Minimal inhibitory concentration
MLST: Multilocus sequence type
mpf: Mating pair formation
NAL: Nalidixic acid
NRL AR: National Reference Laboratory for Antimicrobial Resistances
OMQR: Plasmid-mediated quinolone resistance
ORF: Open reading frame
*oriT*: Origin of transfer
PCR: Polymerase chain reaction
PFGE: Pulsed-field gel-electrophoresis
QRDR: Quinolone resistance-determining regions
SMX: Sulfamethoxazole
SAC: Sodium acid
ST: Sequence type
TA: Toxin-antitoxin
TAZ: Ceftazidime
TET: Tetracycline
TMP: Trimethoprim
UK: United Kingdom
US: United States
ZoMo: Zoonosis monitoring
